# Identification of disease-causing genes using microarray data mining and Gene Ontology

**DOI:** 10.1186/1755-8794-4-12

**Published:** 2011-01-26

**Authors:** Azadeh Mohammadi, Mohammad H Saraee, Mansoor Salehi

**Affiliations:** 1 Intelligent Databases, Data mining and Bioinformatics Laboratory, Isfahan University of Technology, Isfahan, Iran; 2 Dept. of Genetics, Medical School, Isfahan University of Medical Sciences, Isfahan, Iran

## Abstract

**Background:**

One of the best and most accurate methods for identifying disease-causing genes is monitoring gene expression values in different samples using microarray technology. One of the shortcomings of microarray data is that they provide a small quantity of samples with respect to the number of genes. This problem reduces the classification accuracy of the methods, so gene selection is essential to improve the predictive accuracy and to identify potential marker genes for a disease. Among numerous existing methods for gene selection, support vector machine-based recursive feature elimination (SVMRFE) has become one of the leading methods, but its performance can be reduced because of the small sample size, noisy data and the fact that the method does not remove redundant genes.

**Methods:**

We propose a novel framework for gene selection which uses the advantageous features of conventional methods and addresses their weaknesses. In fact, we have combined the Fisher method and SVMRFE to utilize the advantages of a filtering method as well as an embedded method. Furthermore, we have added a redundancy reduction stage to address the weakness of the Fisher method and SVMRFE. In addition to gene expression values, the proposed method uses Gene Ontology which is a reliable source of information on genes. The use of Gene Ontology can compensate, in part, for the limitations of microarrays, such as having a small number of samples and erroneous measurement results.

**Results:**

The proposed method has been applied to colon, Diffuse Large B-Cell Lymphoma (DLBCL) and prostate cancer datasets. The empirical results show that our method has improved classification performance in terms of accuracy, sensitivity and specificity. In addition, the study of the molecular function of selected genes strengthened the hypothesis that these genes are involved in the process of cancer growth.

**Conclusions:**

The proposed method addresses the weakness of conventional methods by adding a redundancy reduction stage and utilizing Gene Ontology information. It predicts marker genes for colon, DLBCL and prostate cancer with a high accuracy. The predictions made in this study can serve as a list of candidates for subsequent wet-lab verification and might help in the search for a cure for cancers.

## Background

One of the most important areas of medical research is the identification of disease-causing genes. Identification of these factors can improve the process of diagnosis and the treatment of diseases. It is known that certain diseases, such as cancer, are reflected in the change of the expression values of certain genes. For example, normal cells may become cancerous due to genetic mutations. These changes affect the expression level of genes. Gene expression is the process of transcribing a gene's DNA sequence into RNA. A gene's expression level indicates the approximate number of copies of that gene's RNA produced in a cell and it is correlated with the amount of the corresponding proteins made [1].

The recent advent of microarray technology has made simultaneous monitoring of thousands of gene expressions possible. Analyzing gene expression data can indicate the genes which are differentially expressed in the diseased tissues [2]. The main problem of microarray data is its limited number of samples with respect to number of genes. Many of these genes have no role in creation of the disease of interest; therefore identification of disease-causing genes can determine not only the cause of the disease, but also its pathogenic mechanism. Diagnostic tests and classification of patients can be done by using marker genes, which will reduce laboratory costs and increase the accuracy. Identification and selection of a subset of genes as disease-causing genes is called gene selection.

Different methods have been proposed in the literature for gene selection. They can be organized in three categories: filter methods, wrapper methods and embedded methods [3]. Filter methods evaluate the goodness of the genes looking only at the intrinsic characteristics of the data, based on the relation of each single gene with the class label by the calculation of simple statistical criteria [4]. Some of the methods are parametric and some are nonparametric. Parametric methods have strict assumptions on the analyzed data, including: normal distribution, homogeneous variances between data groups and continuous measures with equal intervals. Non-parametric methods do not require above assumptions, so they are computationally easier and quicker but statistically less powerful [5].

There is a large variety of parametric methods such as Signal to Noise Ratio (SNR) [6] and Fisher [7]. More recently a novel filter method called SDED (Standard Deviation Error Distribution) is proposed which utilizes variations within class and amongst-class in gene expression data [8]. Wilcoxon rank sum test is an example of non-parametric filter methods [9]. Filter methods are fast and simple but they do not consider the correlation of genes and lead to redundancy in the selected gene sets.

In the 'wrapper' approach a search is conducted in the space of genes, evaluating the goodness of each found gene subset by the estimation of the accuracy of the specific classifier to be used, training the classifier only with the found genes [4]. In [4] forward selection is used for gene selection and in [10,11] a genetic algorithm is used to find informative genes. Martinez and co-workers have proposed a swarm intelligence feature selection algorithm based on the initialization and update of only a subset of particles in the swarm [12]. In [13] a hybrid Particle Swarm Optimization (PSO) and Genetic Algorithm (GA) method is used for gene selection. Wrapper methods consider the correlation of genes but they have high computational complexity.

In the embedded methods, the search for an optimal subset of features is built into the classifier construction, and can be seen as a search in the combined space of feature subsets and hypotheses [3]. Guyon et al. [14] proposed an SVM-based learning method, called SVM recursive feature elimination (SVMRFE). In [15] metaheuristic-based methods are used within an embedded approach for gene selection and in [16] random forests method is used for feature selection.

Embedded methods have the advantage that they include the interaction with the classification model, while at the same time being far less computationally intensive than wrapper methods.

Most of the gene selection methods remove irrelevant genes but they do not take the redundancy into account. Redundancy in selected genes increase computational costs and decrease the classification accuracy. Some methods are proposed for redundancy reduction in selected genes. In [17,18] clustering methods are used for determining similar genes and reducing redundancy. In [19] a method called Markov blanket is used for redundancy reduction. Ding and Peng [20] proposed a method called MRMR (Minimum Redundancy Maximum Relevance) which finds a subset of genes which has minimum redundancy and maximum relevance to class, simultaneously.

In [21] a combination of MRMR method and SVMRFE is used for gene selection which takes into account the redundancy among the genes during their selection; also [22] presents a hybrid filter-wrapper feature subset selection algorithm based on particle swarm optimization (PSO) and MRMR.

In this paper, we propose a new framework for gene selection which combines the Fisher filter and the SVMRFE embedded method, with a greedy algorithm to remove the redundant genes.

The microarray data contains valuable information but due to the presence of the noise in the data and the low number of samples, it mostly does not demonstrate the actual genes functionally. This limitation of microarray data can be compensated for, at least to some extent, by using other source of information about genes. In this paper Gene Ontology information is used in addition to gene expression data to determine gene redundancy.

## Methods

### Feature selection using Fisher criteria

In [7], the Fisher criterion score is used for gene selection. It is a filtering method, in which genes are ranked by the equation below:

(1) $$Fisher(g)=\frac{{\left(m_{1}\left(g\right)-m_{2}\left(g\right)\right)}^{2}}{\left(s_{1}^{2}(g)+s_{2}^{2}(g)\right)}$$

where *m*_*1*_*(g) *and *m*_*2*_*(g) *are means of gene *g *expression across the cancerous and normal samples respectively and *s*_*1*_*(g) *and *s*_*2*_*(g) *are standard deviations of gene *g *across the cancerous and normal samples respectively. The Fisher criterion score gives higher values to features whose means differ greatly between the two classes, relative to their variances. Those genes with the highest scores are considered as the most discriminatory genes.

### Feature selection using SVMRFE algorithm

SVMRFE implements the strategy of backward feature elimination. Backward feature elimination methods start with all features and then, on each iteration, the least important feature is identified and removed according to a ranking criterion and the process repeats with remaining features, until the stop criterion is satisfied.

In SVMRFE the ranking criterion is *(W_i_)^2^*, where *W*_*i *_is the corresponding weight of feature *i*. It iteratively trains new SVM and eliminates the features whose corresponding absolute weight is the smallest from the dataset.

Support Vector Machine (SVM), a supervised machine learning technique, is robust against sparse and noisy data and has been shown to perform well in multiple areas of biological analysis including microarray data expression evaluation [23].

Given a training set belonging to two classes, a linear SVM finds an optimal separating hyper plane with the maximum margin between samples of two classes, by solving the following optimization problem:

(2) $$\begin{array}{l} Min\frac{1}{2}W^{T}W\\ subject\;to:y_{i}(W.X_{i}+b)-1\geq 0 \end{array}$$

where *X*_*i *_and *y*_*i *_are i^th ^sample and its label.

This optimization problem can be solved by below Lagrangian function:

(3) $$\begin{array}{l} L(W,b,\alpha )=\\ \frac{1}{2}W\cdot W-\sum_{i=1}^{S}\alpha_{i}(y_{i}(W\cdot X_{i}+b)-1) \end{array}$$

In above equation, *s *shows the number of training samples and *α*_*i *_denotes Lagrange multipliers.

B y differentiating *L *with respect to *W *and *b*, the following equations are obtained:

(4) $$W=\sum_{i=1}^{S}\alpha_{i}y_{i}X_{i}$$

(5) $$\sum_{i=1}^{S}y_{i}\alpha_{i}=0$$

The weight vector *W *is a linear combination of the training samples. Most weights *α*_*i *_are zero. The training samples with non-zero weights are the support vectors [24].

SVMRFE uses the weight vector of SVM as ranking criteria. The pseudo code of the SVMRFE algorithm is as follows [14]:

1. Initialize the dataset to contain all the features.

2. Train the SVM with a linear kernel function on the dataset and get the weight vector

3. Rank the features by the values of.

4. Remove the feature with the smallest value.

5. If more than one feature remains, return to step 2.

Finally the subset of genes which has achieved the highest accuracy of classification is selected as disease's marker genes [14].

It should be mentioned that although in SVMRFE, one gene is removed in each step, the interaction between genes is considered. The weak point of this method is its large amount of computation. To speed up the algorithm, more than one gene can be removed in each step [25].

### Gene Ontology

The Gene Ontology (GO) project is a collaborative effort to address the need for consistent descriptions of gene products in different databases. GO provides a structured controlled vocabulary of gene and protein biological roles, which can be applied to different species. It comprises three hierarchies that define functional attributes of gene products: Molecular Function (MF), Biological Process (BP), and Cellular Component (CC) [26]. The Gene Ontology information can be obtained from [27].

### Semantic similarity measure

Semantic similarity measures can be used to calculate the similarity of two concepts organized in ontology. The semantic similarity of two concepts is calculated based on their information content (IC). The information content of a concept is inversely proportional to its frequency in a corpus. The frequency of a concept *c *, *Freq (c)*, can be defined as the number of times that *c *and all its descendants occur [28]. An estimate for the likelihood of observing an instance of a concept *c *is:

(6) $$Prob(c)=\frac{Freq(c)}{max\;Freq}$$

where *maxFreq *is the maximum frequency of all concepts.

The information content of a concept *c *is defined as (7):

(7) IC=−log(Prob(c))

Semantic similarity measures assume that the similarity between two concepts is related to the extent to which they share information. Given two concepts *c*_*1 *_and *c*_*2*_, their shared information, *Share (c*_*1*_*, c*_*2*_), can be defined as the information content of their most informative common ancestor (*CommonAnc(c*_*1*_*,c*_*2*_)) [28]:

(8) $$\begin{array}{l} Share(c_{1},c_{2})=\\ max\{IC(a)|a\in CommonAnc(c_{1},c_{2})\} \end{array}$$

The most common semantic similarity measures are Resnik [29],Lin [30] and Jiang [31].

## Results and Discussion

### Proposed method

As mentioned previously, filtering methods are amongst the most common methods for gene selection. These methods have low computational complexity and so can be used easily in large, high dimensional datasets such as microarrays; but these methods evaluate the discriminative power of each gene separately and the interaction of genes are ignored. Also these methods do not take into account the correlation among genes and so the selected gene set may have redundancy. SVMRFE is one of the most successful gene selection methods because it considers the interaction of genes and it can remove irrelevant feature using its ranking criterion. However it is not able to remove redundant features because if one of the features has a large weight, the redundant feature will also get a large weight, but according to recursive feature elimination algorithm, only the features with small weight are removed; therefore the final gene set may have redundancy [32,33].

In this paper we have proposed a combined approach which utilizes the advantages of the filter and embedded methods and which incorporates a method for reducing redundancy in the selected genes. The stages of proposed method are:

1. Ranking genes using the Fisher criterion;

2. Redundancy reduction by considering expression and semantic similarity;

3. Selection of final gene set using SVMRFE method.

### Redundancy reduction by considering expression and semantic similarity

Redundant genes increase the computational time and decrease the accuracy; so in the proposed method, after filtering genes using the Fisher criterion and before applying the SVMRFE method, we reduce redundancy by considering the similarity of the genes. When there are sufficient samples, similar genes can be determined based on Pearson correlation coefficient which considers the linear correlation and is computed based on gene expression levels. Due to the low number of samples in microarray experiments, computing the similarity of two genes based only on their expression value cannot be very accurate. On the other hand, the similarity of genes can be determined from information about their involvement in biological processes and functions. There is some such information available in the Gene Ontology (GO) but it is incomplete, e.g., information for some genes is missing from GO. Therefore GO alone cannot be used to determine the similarity of genes.

In the proposed approach, gene expression values and information from GO are combined to determine the similarity of genes. The similarity measure used is the average of the expression similarity and the semantic similarity:

(9) $$\begin{array}{l} S(g_{i},g_{j})=\\ \frac{S_{exp}(g_{i},g_{j})+S_{sem}(g_{i},g_{j})}{2} \end{array}$$

where *S_sem _(g_i_, g_j_) *and *S_exp _(g_i_, g_j_) *are the semantic similarity and the expression similarity of *g*_*i *_and *g*_*j*_, respectively. The expression similarity of two genes is computed based on the Pearson correlation coefficient:

(10) $$\begin{array}{l} S_{exp}(g_{i},g_{j})=\\ \frac{\sum_{k=1}^{d}\left(g_{ik}-{\bar{g}}_{i}).(g_{jk}-{\bar{g}}_{j}\right)}{\sqrt{{\left(g_{ik}-{\bar{g}}_{i}\right)}^{2}}.\sqrt{{\left(g_{jk}-{\bar{g}}_{j}\right)}^{2}}} \end{array}$$

where ${\bar{g}}_{i}$ is the average value of gene *g*_*i *_expressions and *g*_*ik *_is the value of k^th ^sample in gene *g*_*i*_.

The semantic similarity of two genes is computed based on Lin's similarity criteria [30], which is defined as below:

(11) $$Sim_{Lin}=\frac{2*Share(c_{1},c_{2})}{IC(c_{1})+IC(c_{2})}$$

In the proposed method, a greedy approach has been used in the second stage, to reduce redundancy by removing similar genes, which utilizes (9) as similarity measure.

The greedy algorithm takes as input, from first stage, a list of 500 genes sorted in ascending order of discriminative power. By selecting the first gene of this list, we can ignore other similar genes, because they have less discriminatory power. Genes whose similarity measure to the first gene exceeds a threshold are removed from the list. Next the second gene in the list is selected and again similar genes to it are deleted from the list. This process will be repeated to the end of the list. The remaining genes in this stage are those that have good discriminatory power and their pairwise similarities are below a determined threshold. The greedy approach's pseudo code is shown below:

Inputs:

List of ranked genes: *L *= [*g*_*1*_,*g*_*2*_, ..., *g*_*k*_]

Threshold: *ts*

Similarity matrix: *S*

Initialize:

*RL *= []

*i *= 1

Repeat until *L *= []

% Select the top ranked genes from *L*

% and add it to *RL*

*RL *= [*RL, L(i)*]

*g *= *L(i)*

% eliminate the i^th ^feature from *L*

*L*(:,*i*)=[]

For *j *= *(i+1)*:length(*L*)

If s(*g,L(j)*)≥ *ts*

*L*(:,j) =[]

End

*i *= *i*+1

outputs: Reduced list of genes: *RL*

The threshold is a number between 0 and 1. The smaller the threshold, the more genes will be removed but this can lead to losing some informative genes. On the other hand, when threshold is close to 1, few genes are removed which can lead to some redundant genes remaining. To determine an appropriate threshold we varied the threshold value between 0.1 and 0.95 at intervals of 0.05. The best threshold value is the one which leads to highest accuracy on average. Applying this empirical approach to the datasets, resulted in the threshold being set to 0.8.

### Datasets

In order to evaluate the proposed gene selection method, following datasets are analyzed.

### Colon cancer dataset

In this dataset, expression levels of 40 tumor and 22 normal colon tissues for 6,500 human genes are measured using the Affymetrix technology. A selection of 2,000 genes with highest minimal intensity across the samples has been made in [34]. The dataset we have used is available at [35]. The data is preprocessed by carrying out a base 10 logarithmic transformation and standardizing each sample to zero mean and unit variance.

### DLBCL dataset

Diffuse Large B-Cell Lymphoma (DLBCL) is the most common subtype of non-Hodgkin's lymphoma. Alizadeh et.al have shown that there is diversity in gene expression among the tumors of DLBCL patients, reflecting differentiation state of the tumor [36]. The dataset we have used in this paper is available at [37]. This dataset consists of 40 samples that have information about overall survival, and each sample has expression profile of 4026 genes. By setting threshold value of overall survival at 4 years, data of prognosis can be categorized into two groups. 19 samples were alive after 4 years and 21 of them lived shorter.

The ratio values in the dataset were log -transformed so there is no need to carry out logarithmic transformation, but this dataset has missing value. We have imputed the missing values by FCM-GO-impute method [38]; also all the sample vectors in the dataset are normalized in order to have the zero mean and standard deviation of one.

### Prostate dataset

The prostate dataset was first published in [39]. This dataset was created using Affymetrix platform and provides the expression levels of 12,600 genes for 50 normal tissues and 52 prostate cancer tissues. The dataset is available at [40]. The original dataset is normalized so that each sample vector has 0 for mean and 1 for standard deviation.

### Experimental results

After preprocessing of data, we applied the proposed method on 3 public datasets to identify the most discriminatory genes; also we compared the proposed method with some other common methods. In order to be able to compare the results, in all gene selection methods, support vector machines with linear kernel function is used for classification step.

The performance of different methods is measured in terms of accuracy, sensitivity and specificity of classifier. Accuracy is the fraction of correctly classified samples over all samples.

(12) $$Accuracy=\frac{TN+TP}{TN+FN+FP+TP}$$

where *TP*, *TN*, *FP*, and *FN*, stand for the number of true positive, true negative, false positive and false negative samples. A true positive (*TP*) is a positive sample which is predicted correctly as positive. A true negative (*TN*) is a negative sample which is predicted correctly as negative. A false positive (*FP*) occurs when the outcome is incorrectly predicted as positive when it is actually negative. A false negative (*FN*) occurs when the outcome is incorrectly predicted as negative when it is actually positive.

Sensitivity is the fraction of the real positives that are correctly predicted as positives and specificity is the fraction of the real negatives that are correctly predicted as negatives. Sensitivity and specificity describe how well the classifier discriminates between case with positive and with negative class.

(13) $$Sensitivity=\frac{TP}{TP+FN}$$

(14) $$Specificity=\frac{TN}{TN+FP}$$

Due to the limited number of samples, performance is evaluated using 10 fold cross-validation. The data is randomly divided into 10 non-overlapping partitions of approximately equal size. Each one is used in turn for testing while the remainder is used for training, i.e., 9/10 of data is used for training and 1/10 for testing. This is repeated 10 times. The overall error rate is the average of error rates on each partition.

In some papers, such as [14,18], the feature selection process is applied to the entire set of data and cross-validation is only applied on the classifier construction process, not taking into account feature selection. This approach is referred to as internal cross-validation. Using cross-validation in this manner leads to selection bias, as the feature selection would not be based on the particular training set for each cross validation (CV) run. Hence, overly optimistic error rates would be obtained. To prevent this selection bias from occurring, an external cross-validation process should be implemented following the feature selection at each CV stage, that is, the feature selection is performed based only on those samples set aside as training samples at each stage of the CV process, external to the test samples at each stage [41].

### Statistical analysis

In continue the result of applying the proposed method is presented and it is compared by some other conventional methods based on accuracy, sensitivity and specificity of the classifier which is built on selected genes. Table 1, Table 2 and Table 3 show the average accuracy, average sensitivity and average specificity of different methods respectively. In each of these tables the number of selected genes in each method is shown as well.

**Table 1:** Comparison of different gene selection methods based on accuracy

Methods										
**dataset**	**M1**	**M2**	**M3**	**M4**	**M5**	**M6**	**M7**	**M8**	**M9**	**M10**

Colon	86.1	86.8	89.9	91.6	89.7	91.8	93.3	91.2	93.2	94.7

DLBCL	89.2	89.6	91.9	93.7	92.5	94.4	95.8	93.6	95.4	96.8

Prostate	80.7	91.1	92.8	93.1	92.2	93.8	94.2	93.8	95.1	95.9

**M1 = No-sel**: classification without gene selection

**M2 = Fisher**: classification after using Fisher criteria for gene selection

**M3 = Fisher-R**: classification after using Fisher criteria and redundancy reduction greedy approach for gene selection

**M4 = Fisher-RG**: classification after using Fisher criteria and redundancy reduction greedy approach considering Gene Ontology information for gene selection

**M5 = SVMRFE**: classification after using SVMRFE algorithm

**M6 = SVMRFE-R**: classification after using SVMRFE algorithm and redundancy reduction greedy approach for gene selection

**M7 = SVMRFE-RG**: classification after using SVMRFE algorithm and redundancy reduction greedy approach considering Gene Ontology information for gene selection

**M8 = Fisher-SVMRFE**: classification after using combination of Fisher criteria and SVMRFE algorithm for gene selection

**M9 = Fisher-R-SVMRFE**: classification after using proposed framework without considering Gene Ontology for gene selection

**M10 = Fisher-RG-SVMRFE**: classification after using proposed framework for gene selection

**Table 2:** Comparison of different gene selection methods based on sensitivity

Methods										
**dataset**	**M1**	**M2**	**M3**	**M4**	**M5**	**M6**	**M7**	**M8**	**M9**	**M10**

Colon	82.1	81.9	84.9	86.6	84.5	86.8	88.4	86.4	88.5	90.1

DLBCL	85.3	83.4	87.2	89.0	87.1	89.2	91.0	88.3	91.1	92.6

Prostate	82.1	83.1	88.9	89.4	88.4	91.7	92.3	90.1	92.4	93.5

**Table 3:** Comparison of different gene selection methods based on specificity

Methods										
**dataset**	**M1**	**M2**	**M3**	**M4**	**M5**	**M6**	**M7**	**M8**	**M9**	**M10**

Colon	80.2	78.9	81.8	83.6	81.6	83.7	85.3	83.4	85.4	87.0

DLBCL	83.4	82.8	85.7	88.7	85.5	87.9	90.7	87.3	89.4	91.5

Prostate	81.8	82.9	88.8	89.1	88.6	92.3	93.1	89.9	93.2	94.1

As Table 1 shows, the classifier performance is improved when a gene selection method is applied before classification, with the exception of the Fisher method which uses the Fisher ranking to select the informative genes. As Table 2 and Table 3 show, the sensitivity and specificity of the classifier is reduced when the Fisher method is used in the gene selection step, as compared with case where we have not used gene selection method before classification. Table 4 shows the number of selected genes in different methods.

**Table 4:** Number of selected genes in different gene selection methods

Methods										
**dataset**	**M1**	**M2**	**M3**	**M4**	**M5**	**M6**	**M7**	**M8**	**M9**	**M10**

Colon	2000	67	27	23	27	25	23	26	15	12

DLBCL	4026	56	29	28	36	25	23	27	18	15

Prostate	12600	102	32	28	82	31	27	56	21	14

Comparing the results of Fisher-R (using the Fisher criteria and the redundancy reduction greedy approach for gene selection) and SVMRFE-R (using the SVMRFE algorithm and the redundancy reduction greedy approach for gene selection) with Fisher and SVMRFE respectively, demonstrates that reducing the redundancy of selected genes, leads to a better performance. Comparing Fisher-RG (using the Fisher criteria and the redundancy reduction greedy approach with GO information) and SVMRFE-RG (using the SVMRFE algorithm and the redundancy reduction greedy approach with GO information for gene selection) with Fisher-R and SVMRFE-R, shows that using GO information in addition to the gene expression data, can have a positive effect. Our proposed method (Fisher-RG-SVMRFE) has a better performance than any of the other methods, in terms of accuracy, sensitivity and specificity.

Table 5 lists the published results on the colon cancer dataset. Comparing the results of our proposed framework with the results presented in this table shows that the accuracy of our method is higher than that for the other methods, except [14]. However, it should be mentioned that in [14], the feature selection process is applied to the entire set of data and cross-validation is applied on the classifier construction process. In other words, it suffers from the selection bias problem discussed earlier. This problem exists in [18] as well.

**Table 5:** The reported results for colon cancer dataset in some papers

Ref	[14]	[18]	[11]	[46]	[20]	[23]	[43]
Accuracy	98.0	91.9	93.0	92.0	93.6	90.3	88.8

Number of selected genes	4	3	15	-	10	-	10

Table 6 shows the results of some papers which have studied DLBCL dataset. Comparison the results of our proposed framework with the results presented in this table shows that the accuracy obtained from our method is better than the results obtained in other methods, except [42] which suffers from the selection bias problem.

**Table 6:** The reported results for DLBCL cancer dataset in some papers

Ref	[10]	[42]	[45]	[44]
Accuracy	93.8	99.0	95.0	93.0

Number of selected genes	10	7	5	14

### Biological analysis

In addition to the evaluation of the gene selection method based on the classification performance, the selected genes were analyzed from a biological view. This revealed that there is an overlap between the gene set selected by the proposed method and genes which are predicted as marker gene for DLBCL or colon cancer in other articles. In addition, the study of the molecular function of the genes selected by our method strengthened the hypothesis that these genes are involved in the process of cancer growth and progression.

Table 7 lists the genes predicted by our method to be colon cancer markers. Seven of these (highlighted with a * in Table 7) were also reported in [43]. Six of genes in Table 7 (*NDP kinase*, *Complement factor D*, *Collagen alpha 2 (XI), Desmin*, *Myosin *and *CD37*) are recognized as marker genes for colon cancer in [11]. These genes are marked with + in Table 7.

**Table 7:** List of selected genes for colon cancer using proposed framework

Genes name
Human putative NDP kinase (nm 23 - H2S) mRNA +
Human p58 natural killer cell receptor precursor mRNA
Human cysteine - rich protein (CRP) gene, exons 5 and 6*
Complement factor D precursor (H. sapiens) + *
Human cell adhesion molecule (CD44) mRNA*
H.sapiens mRNA for GCAP - II/uroguanylin precursor*
Collagen alpha 2(XI) chain (H. sapiens) +*
INTEGRIN ALPHA - 6 PRECURSOR (Homo sapiens)
Human desm in gene +*
Myosin regulatory light chain 2 +*
TRANSCRIPTION FACTOR ATF - A AND ATF - A - DELTA (Homo sapiens)
LEUKOCYTE ANTIGEN CD37 (Homo sapiens) +

With due attention to molecular function of selected genes it seems that our proposed method has been able to select a set of cancer-related genes. For example, gene *p58 *is a natural killer cell receptor and it is thought to play an important role in regulation of the immune response and cell death, so the incorrect function of this gene may cause to cancer growth. The protein encoded by *CD44 *is a cell-surface glycoprotein involved in cell-cell interactions, cell adhesion and migration and its role in tumour metastasis is proved. *GCAP-II *is a key component of several intracellular signal transduction pathways and it can have affective role in cancer growth too [27].

Table 8 presents the list of genes which are selected by our proposed method as DLBCL markers. Among these genes, 6 are overlapped by selected genes in [44] (these genes are marked with * in Table 8); also *E2F-3 *and *JNK3 *are reported in [45] as DLBCL markers (these genes are marked with + in Table 8). *E2F-3 *is a transcription factor and its role in cancer growth is reported in papers [45].

**Table 8:** List of selected genes for DLBCL cancer using proposed framework

Genes name
JAW1 = lymphoid - restricted membrane protein*
E2F - 3 = pRB - binding transcription factor +
erk3 = extracellular signal - regulated kinase 3
JNK3 = Stress - activated protein kinase +
Unknown UG Hs. 120716 ESTs*
Unknown UG Hs. 136345 ESTs*
myosin - IC
receptor r - 1 BB lig and
Unknown UG Hs. 105261 EST*
Receptor protein - tyrosine kinase
thymosin beta - 4
Unknown UG Hs. 169565 ESTs*
Id1 = Inhibitor of DNA binding 1, dominant negative helix - loop - helix protein
Unknown UG Hs. 124922 ESTs*
Unknown Hs. 33431 ESTs

Considering molecular function of erk3, *JNK3 and Receptor protein-tyrosine*, strengthen the hypothesis that they can be cancer-related genes because Kinase family genes are the main components of signal transduction system and variation in their expressions may cause cancer growth.

## Conclusions

One of the most important applications of microarrays is the identification of disease causing genes. The selection of these genes is accomplished by analysis of gene expression data in different samples. However the application of microarrays for this purpose is limited by the fact that gene expression data may be incorrect or unknown.

In addition, because of the limited number of samples, gene expression datasets do not represent the real distribution of data. Therefore, to reduce these problems, in this paper, gene expression data is supplemented with a reliable source of information on genes, namely the Gene Ontology (GO).

The proposed approach has three stages:

1. Ranking the genes using the Fisher criterion.

2. A greedy redundancy reduction that utilizes information from GO, in addition to gene expression levels.

3. Selection of final set of predicted disease marker genes based on the SVMRFE algorithm.

We have utilized the advantages of both filtering methods and embedded methods. In stage 1, a significant number of irrelevant genes are removed using the high-speed Fisher filtering method. Since filtering methods do not take into account the correlation amongst genes, the remaining genes will still have a large amount of redundancy. In order to reduce this redundancy, a greedy approach has been proposed for removing similar genes. This approach calculates the similarity between genes using both information from GO and gene expression data. Finally genes that remain after this stage are processed more accurately by the SVMRFE method to derive the disease marker genes.

The results of applying the proposed method on the colon, DLBCL and prostate cancer datasets showed that this method can improve classification performance.

In addition to classification performance, the final gene set has been evaluated from a biological view. This has strengthened the hypothesis that the selected genes may have a significant role in cancer. The final approval of these genes as cancer factors will require more biological and laboratory investigations. The predictions made in this study can serve as a list of candidates for subsequent wet-lab verification and might help in the search for a cure for colon, DLBCL or prostate cancer. It should be mentioned that the final approval of these genes as cancer factors, require more biological and laboratory investigations and selected genes, can only provide genetic researchers some clues for more research.

## Competing interests

The authors declare that they have no competing interests.

## Authors' contributions

AM and MHS conceived of the study and participated in the design of and implementation of algorithms. AM performed the statistical analysis, and drafted the manuscript. MHS participated in its coordination and refined the manuscript. MS analyzed the biological results and refined the manuscript. All authors provided critical input during manuscript preparation and read and approved the final manuscript.

## Pre-publication history

The pre-publication history for this paper can be accessed here:

http://www.biomedcentral.com/1755-8794/4/12/prepub
